# Unusual variant of Cantrell's pentalogy?

**DOI:** 10.4103/1817-1737.41916

**Published:** 2008

**Authors:** Basant Kumar, S. B. Sharma, Deepak K. Kandpal, L. D. Agrawal

**Affiliations:** *Department of Pediatric Surgery, Sir Padampat Mother and Child Health Institute (JayKayLon Hospital), SMS Medical College, Jaipur, India*

**Keywords:** Cantrell's pentalogy, chest wall defect, congenital diaphragmatic hernia

## Abstract

A 12-hour-old male infant presented with prolapsed abdominal content through a defect on left side of chest wall with respiratory distress. A thorough clinical examination suggested absence of ectopia cordis, abdominal wall defect, and any bony anomaly. The child expired after 6 hours of admission because of respiratory distress and electrolyte imbalance. Is congenital defect of chest wall associated with diaphragmatic hernia without ectopia cordis and omphalocele, an unusual variant of Cantrell's pentalogy?

## Case Report

A 12-hour-old, full-term, home-delivered male weighing 2.25 kg was admitted to our institute with complaints of difficulty in breathing and protruded swelling on left side of chest wall since birth. This was his mother's first pregnancy, during which there had been no history of recognized infections or unusual environmental exposure. In the family history, there were no instances of congenital defects. The neonate was resuscitated, kept on ventilator, and broad-spectrum antibiotics were administered.

**Figure 1:**
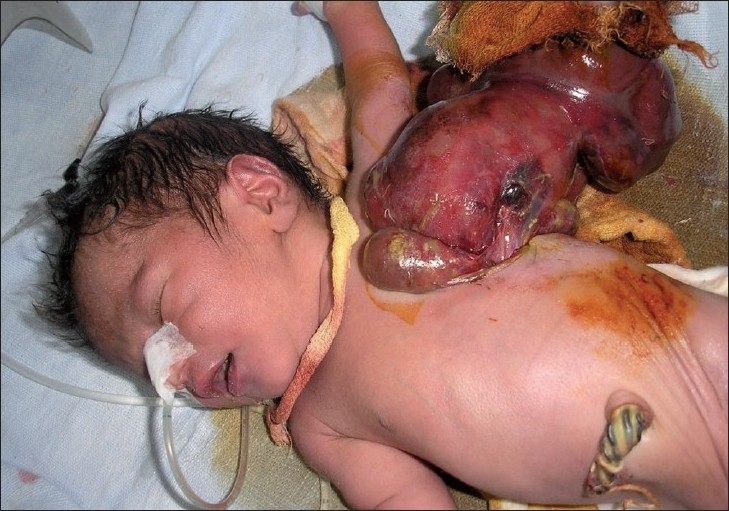
Chest wall defect with prolapsed abdominal contents

**Figure 2:**
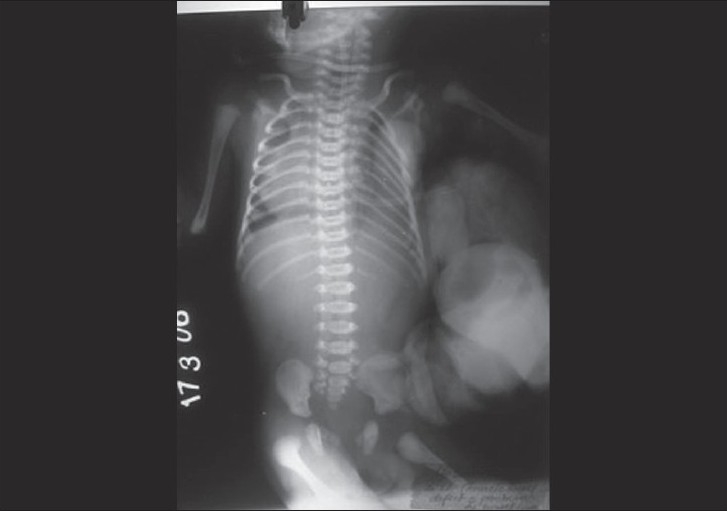
Radiograph showing mediastinal shift and NG tube in stomach lying outside the chest wall

On examination, the baby had severe respiratory distress with peripheral cyanosis. Chest movement on left side was absent, and apex beat was seen on right side near midclavicular line at fourth intercostal space. The protruded swelling present on left side of chest wall appeared as having bowel loops with it [Figure 1]. A defect of about 3 × 4 cm in 4^th^/5^th^ intercostal space was felt on left side of chest wall, through which sac was protruding containing stomach, small and large bowel, spleen, and part of liver. Overlying sac was ruptured. On auscultation, heart sound was heard on right side of chest without any appreciable murmur or added sound with decreased air entry at inframammary region. No air entry was found on left side of chest. On x-ray chest, mediastinum was found to be shifted on the right side without any bony deformity [Figure 2]. ECG was normal. Laboratory findings suggested that the baby was in severe respiratory acidosis with hypercarbia and electrolyte imbalance. The baby died before any surgical intervention in spite of all our medical efforts.

## Discussion

Cantrell, Haller, and Ravitch, in (1958), described a syndrome characterized by a midline supraumbilical abdominal wall defect, a defect of the lower sternum, a deficiency of anterior diaphragm, a defect in the diaphragmatic pericardium, and congenital intracardiac defects.[1] This syndrome is extremely rare and the complexity of the syndrome is incompatible with life, so the exact incidence could not be found. However, an incidence of 1:100,000 births and male predominance (M:F ratio, 2:1.2) have been described in literature in developed countries.[2] On the basis of embryological development, this syndrome may be classified in two groups.[1] The first group would appear to arise as a result of developmental failure of a segment of the mesoderm and comprises 3 defects, viz., diaphragmatic, pericardial, and intracardiac lesions. The second group includes the sternal wall defect and the abdominal wall defect, both of which appear to arise due to failure of migration of the paired primordial structures. Many variants of Cantrell's pentalogy have been described in view of the postulated embryological development of these defects[3,4] and classified[2] as:

Class 1: Exact diagnosis, with the 5 present defects
Class 2: Probable diagnosis, with 4 defects (including intracardiac and abdominal wall defects)
Class 3: Incomplete diagnosis, with combination of the defects (always accompanied by sternal defects)

In our patient, there was no abdominal wall defect and sternum appeared normal with no appreciable cardiac anomaly; but diaphragmatic hernia, along with chest wall defect, was present. As the baby was not explored, we could not opine about pericardial defects.

## Is it a variant of cantrell's pentalogy?

In literature, we could not find any such type of case presented with congenital diaphragmatic hernia with prolapsed bowel through chest wall defect. It may or may not be a variant of Cantrell's pentalogy but seems to be related with it, and we think that it is a unique congenital anomaly, not mentioned in literature till now and needs documentation. The probable explanation of chest wall defect is due to failure of ventral migration of myotomes in this region so that normal differentiation into the various muscle layers at the chest wall does not occur, similar to omphalocele. Pressure effect over chest wall because of prolapsed abdominal content into chest cavity through diaphragmatic defect further aggravates this condition.

## References

[1] Cantrell JR, Haller JA, Ravitch MM (1958). A syndrome of congenital defects involving the abdominal wall, sternum, diaphragm, pericardium and heart. Surg Gynecol Obstet.

[2] Lopez JA, Lopez AG, Leon IH (2004). Presentation and discussion of a patient with pentalogia of Cantrell. Cuban Rev Obstet Ginecol.

[3] Sachis-Wall-plate L, Beltra-Tip R (1992). Cantrell's pentology: Complete treatment, step by step. Cir Pediatr.

[4] Chen LJ, Wu JM, Yang YJ, Wang JN, Lin CS (1997). Cantrell's syndrome in an infant. J Formos Med Assoc.

